# On Modern Progress in Ophthalmic Medicine and Surgery

**Published:** 1895-04-13

**Authors:** Robert Brudenell Carter

**Affiliations:** Consulting Ophthalmic Surgeon to St. George's Hospital


### ON MODERN PROGRESS IN OPHTHALMIC MEDICINE AND SURGERY.

By ROBERT BRUDENELL CARTER, F.R.C.S., Consulting Ophthalmic Surgeon to St. George's Hospital.

### DISEASES OF THE CORNEA.

(*Continued from page* 417, *Vol. XVII.*)

THE treatment of the more serious forms of ulceration of the cornea has been greatly modified by modern knowledge of the presence and potentialities of pathogenic microbes, although cases are frequently encountered in which the resistance to healing appears to depend, primarily at least, upon defective innervation. It was long ago pointed out by Schiff that destructive ulceration of the cornea might be produced in rabbits by section of the trunk of the fifth nerve; and his experiments led him to the conclusion that the result was partly due to the withdrawal of nervous influence from the nutritive operations of the affected tissue, and partly to increased liability to injury arising from the loss of protective sensations. If the edges of the lids were pared and united by suture, and so kept until the nerve function was restored, ulceration of the cornea seldom or never occurred. In the conduct of all such experiments, however, there are many sources of error; but it is well known as a clinical fact that ulceration of the cornea is prone to occur in conditions of extreme exhaustion, and also that it is liable to occur symmetrically, in corresponding positions in both eyes, in circumstances which leave no doubt of its dependence upon diminished or perverted nervous influence.

The gravity of a corneal ulcer depends upon its situation, its superficial extent, its depth, and upon the age and general condition of the patient. If it be more than mere loss of epithelium, that is to say if it penetrate to the true corneal tissue, it will leave a cloudy or opaque scar in healing; and this scar, if it cover or encroach upon the pupillary area, must become an impediment to vision. If the ulcer be large and deep, its healing must be attended by cicatricial flattening of the unaffected portions of the membrane, by which the eye may be rendered entirely useless; and if, instead of healing, it should perforate, it will, if large, permit the escape of the lens, and if small, will probably lead to an adhesion between the iris and the cicatrix. Any corneal ulcer, therefore, unless or until it is definitely on the road to recovery, must always be held to require a grave and guarded prognosis. The ulcers which are presumably "neuro-paralytic" are usually of very slow progress, and give an abundance of time for treatment, while those which are septic, whether they are produced by suppuration in the cornea or by injury, are usually both rapid and destructive.

As a typical case of "neuro-paralytic" ulcers I may describe that of a lady who consulted me last year. Her conjunctiva were somewhat flushed, and on the inner side of each cornea, at its margin, there was a crescent-shaped ulcer, extending in length from a quarter to a third of the circumference, about two millimetres wide in the central portion, and tapering off at each extremity. Each ulcer had a clear margin and an uneven or humpy floor, and penetrated, at its deepest part, through about half the thickness of the cornea. The condition had remained practically the same for some months, sometimes one eye a little worse or better, sometimes the other, but the ulcers never healing, and, on the whole, slowly increasing both in length and depth. There was constant discomfort, occasional pain, and, although the sight was scarcely at all affected, there was no power of employing the eyes. Bactericides and stimulants were applied locally, and attention was given to the general health, which, from various causes, had a good deal run down, but very little improvement followed. I saw the patient from time to time, but several months elapsed before she came permanently under my care. I stained the ulcers with fluorescin, so as to define their exact limits, and then, chloroform having been administered, scraped away the whole of the diseased surface with a sharp spoon, undermining the edges, and cutting away the projecting parts with scissors. Each eye was then thoroughly irrigated with an antiseptic solution, the closed lids were covered with a piece of cambric smeared with sanitas vaseline, and over this a pad of cotton wool was secured by strips of plaster. The dressings were not removed for a fortnight, when it was found that one of the ulcers had completely healed, and the other nearly so. With the latter the treatment was repeated, and healing was produced; but only time will show whether the result will be permanent. An ulcer originating in defective nerve action is apt to recur from the continued operation of the cause by which it was produced.

The fluorescin, of which mention is made in the preceding paragraph, is an agent of great value for the purpose there indicated. It is an aniline compound introduced into practice by Dr. von Straub, and it has the effect of staining any denuded portion of the cornea or conjunctiva of a green colour, which on the cornea is much brighter than on the conjunctiva. It is used in a solution containing fluorescin, 1·0; bicarbonate of soda, 2·0; sterilised distilled water, 50·0 parts. A drop or two of this solution is placed within the lids, and the discoloration is produced immediately. It perfectly defines the limits of an ulcer for the purposes of surgical interference, and is also very useful in children, in whom it at once displays the existence of a breach of surface which might not otherwise have been discoverable without an examination under chloroform.

The ordinary septic or sloughing ulcer is frequently the result of injury, attended or followed by septic inoculation. A common form is that produced by some slight accident, as a blow from a twig, or a scratch from an infant's finger nail, the sufferer being often advanced in life. The ulcer is conspicuous, early assuming a grey colour and sloughy aspect, and usually spreads rapidly, both in depth and in extent. If left unchecked, it speedily perforates the cornea, and, as soon as this happens, it is usual for healing to commence. In such a case the corneal tissue is replaced by a layer of effused lymph, which becomes firmly united with the iris, and ultimately forms a cicatrix, which is generally so thin as to yield to the intraocular pressure and become distended, forming what is known as a "staphyloma" of the cornea, and destroying all vision beyond a mere perception of light.

